# Systematic Analysis of Gene Expression Profiles Controlled by hnRNP Q and hnRNP R, Two Closely Related Human RNA Binding Proteins Implicated in mRNA Processing Mechanisms

**DOI:** 10.3389/fmolb.2018.00079

**Published:** 2018-08-30

**Authors:** Sara Cappelli, Maurizio Romano, Emanuele Buratti

**Affiliations:** ^1^Molecular Pathology, International Centre for Genetic Engineering and Biotechnology, Trieste, Italy; ^2^Department of Life Sciences, University of Trieste, Trieste, Italy

**Keywords:** hnRNP Q, Syncrip, hnRNP R, RNA-seq, brain, immune system

## Abstract

Heteregeneous ribonucleoproteins (hnRNPs) are a family of RNA-binding proteins that take part in all processes that involve mRNA maturation. As a consequence, alterations of their homeostasis may lead to many complex pathological disorders, such as neurodegeneration and cancer. For many of these proteins, however, their exact function and cellular targets are still not very well known. Here, we focused the attention on two hnRNP family members, hnRNP Q and hnRNP R, that we previously found affecting TDP-43 activity both in Drosophila melanogaster and human neuronal cell line. Classification of these two human proteins as paralogs is suported by the high level of sequence homology and by the observation that in fly they correspond to the same protein, namely Syp. We profiled differentially expressed genes from RNA-Seq and generated functional enrichment results after silencing of hnRNP Q and hnRNP R in neuroblastoma SH-SY5Y cell line. Interestingly, despite their high sequence similarity, these two proteins were found to affect different cellular pathways, especially with regards to neurodegeneration, such as PENK, NGR3, RAB26, JAG1, as well as inflammatory response, such as TNF, ICAM1, ICAM5, and TNFRSF9. In conclusion, human hnRNP Q and hnRNP R may be considered potentially important regulators of neuronal homeostasis and their disruption could impair distinct pathways in the central nervous system axis, thus confirming the importance of their conservation during evolution.

## Introduction

Regulation of RNA metabolism is an important step in the maintenance of neuronal homeostasis. RNA biosynthesis, editing, and turnover are sustained at different levels by a network of RNA-binding proteins (RBPs) that bind to the pre-mRNA molecule and combinatorially interact with each other. In 2004, a census of all the human proteins known to bind RNA or to be RNA-related identified ~ 1,542 proteins (about 7.5% of all protein-coding genes) as potentially belonging to the RBP family (Gerstberger et al., 2014). This finding also reflects a great importance for these proteins during evolution, as a considerable number of ortholog of these human RBPs was also found in the lower organisms, such as Archea and Bacteria (Anantharaman et al., 2002). Furthermore, bioinformatics analysis of *Saccharomyces cerevisiae* and *Drosophila melanogaster* genomes have also highlighted that 5–8% and 2–3% of genes are predicted to act as RBPs, respectively (Keene, 2001). In addition to the evolutionary conservation the importance of RBPs in the regulation of RNA metabolism is also highlighted by the observation that highly complex tissues, such as brain, express a network of specific RBPs for regulating the RNA homeostasis (e.g., Hu/ELAV family) (De Conti et al., 2016).

The most abundant members of this family are called heterogeneous ribonucleoproteins (hnRNPs) and share several structural and functional properties (Gerstberger et al., 2014). These hnRNP proteins are highly conserved proteins that were originally described as a group of ~20 major factors capable of forming high molecular-weight complexes transiently bound to the nascent heterogeneous nuclear RNA (hnRNA) transcribed by RNA polymerase II (Dreyfuss et al., 1993). Later on, many other proteins involved in controlling RNA processing have been seen to share hnRNP-like features and are now classified as members of this large family, thus including TAR-DNA Binding Protein 43 (TDP-43), CUGBP Elav-Like Family (CELF) proteins, Neuro-Oncological Ventral Antigen (NOVA) proteins, and Fused in Sarcoma (FUS) (Busch and Hertel, 2012). It is important to note that the number of RNA binding proteins capable of altering RNA processing is still growing steadily and a recent attempt at uncovering the number of RBPs that can be produced by HeLa cells has uncovered several hundred putative new proteins for which we still now very little about (Castello et al., 2012). For this reason, it is important to functionally characterize in a systematic manner all the major components of this family.

Structurally, all RBPs contain some common elements (Gerstberger et al., 2014). In particular, hnRNPs contain one or more RNA-binding domains (RBDs) and the majority of them also have arginine-glycine-glycine (RGG) boxes and auxiliary domains, such as acid-rich- and proline-rich domains. Most importantly, many RBPs also present different splicing isoforms and can undergo post-translation modifications as well as nucleocytoplasmic shuttling (Han et al., 2010).

In cells, the equilibrium of hnRNP proteins is finely regulated and alterations in their expression levels can often lead to numerous defects at the level of RNA processing. This can be particularly problematic for neurons that are characterized by a very adaptive and dynamic architecture. As a consequence, perturbation of the neuronal hnRNP levels may lead to neurodegenerative disorders, such as amyotrophic lateral sclerosis (ALS), fronto-temporal lobar dementia (FTLD), spinal muscular atrophy (SMA), and Alzheimer's disease (AD) (Neumann et al., 2006; Vance et al., 2009; Bebee et al., 2012; Berson et al., 2012). Very often, these perturbations are caused by the occurrence of aberrant aggregation of these proteins in the neurons of affected patients (Conlon and Manley, 2017). It is well-established, for example, that aggregation of TDP-43 and FUS in patient brains is a major feature of patients suffering from ALS/FTLD (Neumann et al., 2006; Kwiatkowski et al., 2009; Vance et al., 2009). Further evidences of the relationship between hnRNP and neurodegeneration are also provided by the identification of ALS/FTLD-associated mutations in other hnRNP proteins, including hnRNP A1 and A2/B1 (Kim et al., 2013), likewise to the finding of nuclear and cytoplasmic deposition of hnRNPA3 in the hippocampus of patients with *C9orf72* hexanucleotide expansion mutations (Davidson et al., 2017).

**Figure 1:**
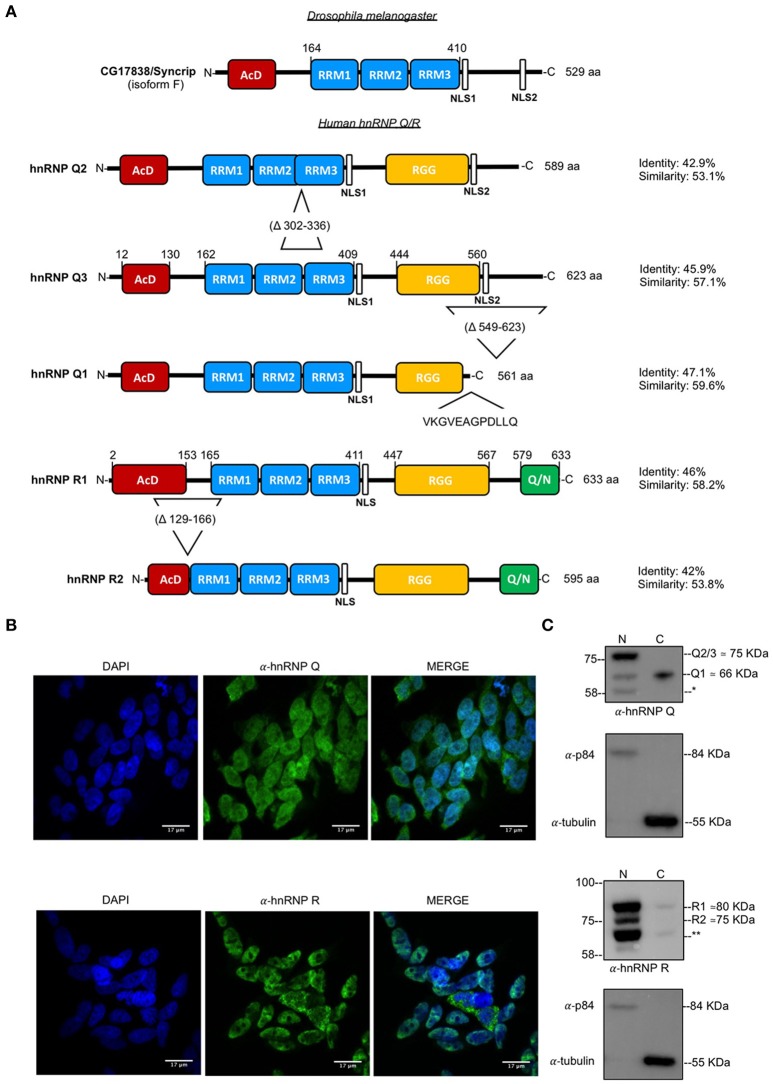
Structure of and subcellular localization of endogenous hnRNP Q and hnRNP R. **(A)** Schematic representation of protein domains and major isoforms of *Drosophila melanogaster* CG17838/Syncrip and human hnRNP Q/ hnRNP R. AcD (acidic domain), RRM (RNA-recognition motif), NLS (nuclear localization signal), RGG (Arg-Gly-Gly)-box and Glutammine/Asparagine (Q/N)-rich domain are highlighted in colored boxes; relative sequence position and amino acids length of each isoform is also reported. *Drosophila melanogaster* CG17838/Syncrip isoform F contains a conserved AcD, three RRM (RRM1, RRM2, and RRM3) and two NLS. Regarding hnRNP Q three major isoforms are represented: hnRNP Q3 is the longest variant and contains an AcD domain, three RRMs, two NLS and an RGG-box; hnRNP Q2 lacks of 36 aa (Δ302-336) between RRM2 and RRM3 compare to the longest variant hnRNP Q3, while hnRNP Q1 lacks of the second NLS and RGG-box region (Δ549-623) from hnRNP Q3 and contains a unique C-terminal domain (VKGVEAGPDLLQ). The isoform 1 of hnRNP R (hnRNP R1) contains an AcD domain, three RRMs, two NLS, an RGG-box and a Q/N-rich domain at C-terminus. The low expressed and neuronal-specific isoform (hnRNP R2) lacking of the 41 aa (Δ129-166) between the AcD domain and the first RRM is also reported. Sequence identity and similarity were calculated using EMBOSS Needle with respect to the *Drosophila melanogaster* CG17838/Syncrip isoform F. We considered this fly isoform, according to its high expression in different stage of life cycle and in all adult tissues, including brain (McDermott et al., 2012). **(B)** Immunofluorescence analysis of the endogenous of human hnRNP Q and hnRNP R (shown in green) in SH-SY5Y cells. Nuclei were visualized using DAPI staining. Scale bars: 17 μm. **(C)** Nuclear and cytoplasmic fractions of endogenous human hnRNP Q and hnRNP R. α-p84 and α-tubulin were used as controls for nuclear and cytoplasmic fractions, respectively. Molecular weight of each isoform is reported. *possible splicing variant of hnRNP Q and ** possible splicing variant of hnRNP R.

Interestingly, from our previous work on this topic, we have observed that major hnRNP cellular proteins can modulate the gain- and loss-of-function effects of one of the major disease players, such as TDP-43 (Mohagheghi et al., 2016; Appocher et al., 2017). In particular, we found that a distinct set of hnRNPs is capable of powerfully rescuing TDP-43 toxicity in the fly eye (Hrb27c, CG42458, Glo, and Syp). From the point of view of RNA metabolism in ALS pathology, among these four proteins, Syp was particularly interestingly because of its well known connections with the nervous system development. In *Drosophila melanogaster*, in fact, this protein was found to regulate the localization of mRNAs driving axis specification and germline formation as well as that of mRNAs involved in the organization of the neuromuscolar junction (McDermott et al., 2012; Mcdermott et al., 2014). Intriguingly, in humans this protein can be found in two well conserved Syp-orthologs, hnRNP Q and hnRNP R (Figure 1), suggesting the occurrence of a progressive functional divergence of these two paralogs in mammalian cells.

The RNA binding protein hnRNP Q, also known as SYNCRIP, was first described in 1997 as a nucleocytoplasmic protein interacting with the synaptotagmin isoform II (Syt-II) C2AB domain in mouse brain lysate (Mizutani et al., 1997) and subsequently found in association with human survival of motor neurons (SMN) gene (Mourelatos et al., 2001). The protein hnRNP Q exists in different splicing isoforms, three of which are the most representative: hnRNP Q3, hnRNP Q2, and hnRNP Q1 (a schematic diagram of each isoform is shown in Figure 1A). The Q3 variant is very similar in sequence (~83% homology) to hnRNP R (Mourelatos et al., 2001) that is also expressed in alternative splicing isoforms, although predominantly as a major isoform known as R1 (UniProt ID O43390-1).

Functionally, hnRNP Q and hnRNP R are already known to regulate different aspects of RNA maturation. More specifically, hnRNP Q was found to promote inclusion of SMN2 exon7 (Chen et al., 2008) and to inhibit C-to-U RNA editing of the apolipoprotein B mRNA (apoB) (Blanc et al., 2001). In addition, this factor can also affect mRNA transport, as demonstrated by the colocalization with ribosomal proteins and other RBPs in neuronal mRNA granules (Bannai et al., 2004; Kanai et al., 2004). Finally, hnRNP Q1 is also able to modulate neuronal morphogenesis and neurite branching in a mouse neuroblastoma cell line by interacting with different mRNAs related to Cdc42 signaling (Chen et al., 2012).

On the other hand, hnRNP R was first described in 1998 in the serum of patients with autoimmunity symptoms (Hassfeld et al., 1998) and subsequntly identified, like hnRNP Q, as a factor bound to the SMN mRNA (Rossoll et al., 2002). At present, it is known that hnRNP R is involved in the transcription and degradation process of *c-fos* mRNA in retinal cells (Huang et al., 2008) and in the expression of immunity factors (Meininger et al., 2016; Reches et al., 2016).

Interestingly, functional rescue of TDP-43 alterations was found to be conserved in the human orthologs of Hrb27c (DAZAP1) and for only one of the human orthologs of Syp (hnRNP Q), but not for the second one (hnRNP R) (Appocher et al., 2017). Based on these results, we have therefore decided to focused the attention on these two members of the hnRNP family whose functions still remain not completely clear. In order to better characterize hnRNP Q and hnRNP R from a neuronal point of view, we have now assessed the cellular localization of these hnRNPs in SH-SY5Y cells and we have investigated changes in the whole transcritome status after their knockdown, looking for gene pathways particularly regulated by these two factors.

## Materials and methods

### Cell culture and gene knockdown

Human neuroblastoma SH-SY5Y cell line (ATCC Microbiology, Manassas, VA) were cultured as described previously (Appocher et al., 2017). To achieve optimal knockdown efficiency, three rounds of silencing were performed on day 1, 2 using Hyperfectamine (Qiagen Inc, Gaithersburg, MD, USA), according to the manufacturer's instruction. The siRNA sense sequences used in this study were as follows: luciferase (siLUC), 5′-uaaggcuaugaagagauac-3′; hnRNP Q (sihnRNP Q), 5′-agacagugaucucucucau-3′; and hnRNP R (sihnRNP R),5′-cauuugggaucuacgucuu-3′.

The mouse motor neuron NSC-34 cell line was cultured in Dulbecco's modified Eagle's medium (DMEM)–Glutamax-I (Gibco- BRL, Life Technologies Inc., Frederick, MD, USA) supplemented with 5% fetal, bovine serum (FBS) (SigmaAldrich, St Louis, MO, USA) and 1% Antibiotic-Antimycotic-stabilized suspension (Sig- maAldrich, St Louis, MO, USA) at 37°C incubator with humidified atmosphere of 5% CO2. Cultures were used 5–15 passages.

For differentiation, NSC-34 cells were seeded to reach 70% confluence the day after and the proliferation medium was exchanged 24 h later to fresh differentiation medium containing 1:1 DMEM/F-12 Ham (SigmaAldrich, St Louis, MO, USA), 1% FBS (SigmaAldrich, St Louis, MO, USA), 1% modified Eagle's medium nonessential amino acids (NEAA) (SigmaAldrich, St Louis, MO, USA), 1% Antibiotic-Antimycotic-stabilized suspension (SigmaAldrich, St Louis, MO, USA) and 1 μM all-trans retinoic acid (RA). Differentiation medium was changed every 2 days and cells were allowed to differentiate for up to 4–7 days.

NSC-34 cells, maintained on proliferation medium (DMEM, 5% FBS, 1% Antibiotic-Antimycotic suspension) represented the undifferentiated control group. Images of undifferentiated (control) and differentiated NSC-34 cells were acquired in light microscopy using a Leica DMIL LED mycroscope equipped with a 20X objective, a Leica DFC450 C camera (Leica Microsystems, Cambridge, UK) and LAS v.4.4.0 Software (Leica application suit). The average length of neurites in the differentiation media was compared to that in the proliferation media and quantified using Fiji NeuronJ (Meijering et al., 2004). Neurite length was analyzed by imaging a minimum of 5 cells per field. The mean of neurite length ± standard error is reported. Statistical significance was calculated using *t-test* (indicated as ^***^ for *P* ≤ 0.001).

### RT-qPCR analysis

Cells were harvested 48 h after the last siRNA transfection and were processed for RT-qPCR analysis. RNA extraction was performed using EuroGOLD TriFast (Euroclone, Milan, Italy), according to the manufacturer's instructions. One Microgram of total RNA was used for the reverse transcription carried out at 37°C using random primers (SigmaAldrich, St Louis, MO, USA) and Moloney murine leukemia virus (M-MLV) Reverse Transcriptase (Gibco-BRL, Life Technologies Inc., Frederick, MD, USA). The resulting cDNA was diluted 1:10 and used for quantitative PCR (qPCR). The target gene sequences were the following: hnRNP Q forward 5′-actgttgaatgggctgatcc-3′, reverse 5′-cctccaagtctttgccattc-3′; hnRNP R forward 5′-gcaaggtgcaagagtccaca-3′, reverse 5′-cacgccagagtacacactgtc-3′; TNF forward 5′-cctctctctaatcagccctctg-3′, reverse 5′-gaggacctgggagtagatgag-3′; ICAM1 forward 5′-ggccggccagctt atacac-3′, reverse 5′-tagacacttgagctcgggca-3′; PENK forward 5′-gtgcagctaccgcctagtg-3′, reverse 5′- tgcaggtttcccaaattttc-3′; TNFRSF9 forward 5′-ttggatggaaagtctgtgcttg-3′, reverse 5′-a ggagatgatctgcggagagt-3′; KLF4 forward 5′-gcggcaaaacctacacaaag-3′, reverse 5′- ccccgtgtgtttacggtagt-3′; KLHL4 forward 5′-ttggagatgatggctgatga-3′, reverse 5′- aagagtttgctctgcgtggt-3′; NRG3 forward 5′-tattcaaaggtggaaaggcatcc-3′, reverse 5′-tgaaggcattcctatggagca-3′; RAB26 forward 5′-tcatctccaccgtaggcatt-3′, reverse 5′-ccggtagtaggcatgggtaa-3′; ARHGA36 forward 5′-ttgaactgacagccacgatg-3′, reverse 5′-gccagactatccacagacac-3′; CT55 forward 5′-atgttgtgactggcaacgtg-3′, reverse 5′-agcaccataaagatggcgag-3′; CARTPT forward 5′- ccgagccctggacatctact-3′, reverse 5′-atgggaacacgtttactcttgag-3′; FOSB forward 5′- accctctgccgagtctcaat-3′, reverse 5′-gaaggaaccgggcatttc-3′; JAG1 forward 5′-atcgtgctgcctttcagttt-3′, reverse 5′-gatcatgcccgagtgagaa-3′; ICAM5 forward 5′-ggctcttcggcctctcag-3′, reverse 5′-gcagttggtgctgcaattc-3′; DUOXA1 5′-ccaagccaaccttcccgat-3′, reverse 5′-cccgatgaataagctggtcac-3′; HMOX1 forward 5′-gccagcaacaaagtgcaag-3′, reverse 5′-gagtgtaaggacccatcgga-3′; KCNAB1 5′-gcaaatcgaccggacagtaac-3′, reverse 5′-gccatgccttggtttatcacat-3′, ACP5 forward 5′-ctacccactgcctggtcaag-3′, reverse 5′-cacgccattctcatcttgc-3′; SDCBP2 forward 5′-ccactacgtgtgtgaggtgg-3′, reverse 5′-tgctcgtagatcacactggg-3′, EFEMP1 forward 5′-cgagcaaagtgaacacaacg-3′, reverse 5′-gatatccaggagggcactga-3′. Housekeeping gene Hypoxanthine phosphoribosyltransferase 1 (HPRT1) and RNA polymerase II subunit A (POLR2A) were used to normalize the results. The sequences of these primers are the following: HPRT1 forward 5′-tgacactggcaaaacaatgca-3′, reverse 5′-ggtccttttcaccagcaagct-3′; RPII forward 5′-gcccacgtccaatgacat-3′, reverse 5′-gtgcggctgcttccataa-3′.

Quantitative PCR reaction was performed in the presence of iQ SYBR green supermix (BioRad, Hercules, CA, USA), using the following conditions for all the target genes, but KLF4 and KLHL4: 95°C for 3 min, 95°C for 10 s, 60°C for 30 s, 95°C for 10 s, 65°C for 1 s. For KLF4 and KLHL4 genes the qPCR conditions were the following: 95°C for 3 min, 95°C for 10 s, 65°C for 30 s, 95°C for 10 s, 65°C for 1 s. The relative gene expression levels were determined using the 2–ΔΔCT method (Schmittgen and Livak, 2008). The mean of relative expression levels ± standard error of three independent experiments is reported. Statistical significance was calculated using *t-test* (indicated as ^*^ for *P* ≤ 0.05, as ^**^ for *P* ≤ 0.01 and as ^***^for *P* ≤ 0.001).

### Immunofluorescence analysis

SH-SY5Y cells (3 × 10^5^) and NSC-34 (3.5 × 10^5^) were plated in 6-well plates containing coverslips. For SH-SY5Y treated with siRNA against hnRNPs and NSC-34 we plated the corresponding number of cells in 6-well plates containing coverslips coated with poly-L-lysine solution at a final concentration of 0.01% (w/v) in H20 (SigmaAldrich, St Louis, MO, USA). After 24 h, cells were washed three times with PBS, fixed in 3.2% paraformaldehyde in PBS for 1 h at room temperature and permeabilized by using 0.3% Triton in PBS for 5 min on ice. Cells were then blocked with 2% BSA/PBS for 20 min at room temperature and immunolabeled with 1:200 rabbit polyclonal antibody anti-hnRNP Q (SigmaAldrich, St Louis, MO, USA) or 1:200 rabbit polyclonal antibody anti-hnRNP R (Abcam, Cambridge, UK) in 2% BSA/PBS overnight at 4°C. Next day, cells were washed three times with PBS, incubated with 1:500 anti-rabbit Alexa-Fluor 488 (Invitrogen, Carlsbad, CA, USA) for 1 h at room temperature and coverslipped with Vectashield-DAPI mounting medium (Vector Laboratories, Burlingame, CA, USA). Each slide was analyzed at the microscopy facility of University of Trieste, using a Nikon Eclipse C1si confocal microscope system mounted on a Nikon TE-2000U inverted microscope with a 60X objective.

### Nuclear and cytoplasmic extraction and western blot analysis

SH-SY5Y cells were seeded in p100 dishes to reach 90% confluence the day of nuclear and cytoplasm extraction. Cells from two dishes were pooled together and the resulting pellets were treated using NER-PER Nuclear and Cytoplasmic Extraction Reagents (ThermoFischer, Waltham, MA, USA) as described in the manufacturer's instructions. Evaluation of the presence/absence of hnRNP Q and hnRNP R in the nuclear and cytoplasm fractions was then carried out by Western blot analysis. Proteins extract (15 μg) for each sample was loaded on a 10% SDS-PAGE gel. The gel was then electroblotted on an Immobilon-P PVDF Membrane (Merck Millipore, Burlington, MA, USA), according to standard protocols and blocked with 4% BSA (SigmaAldrich, St Louis, MO, USA) prepared in 1 × PBS with 0.1% Tween-20 (SigmaAldrich, St Louis, MO, USA). Proteins were incubated with 1:1000 rabbit polyclonal antibody anti-hnRNP Q (SigmaAldrich, St Louis, MO, USA) or 1:1000 rabbit polyclonal antibody anti-hnRNP R (Abcam, Cambridge, UK) and successively were incubated with 1:2000 HRP-conjugated secondary antibody (Dako, Glostrup, Denmark). Protein detections were assessed with Luminata Classico Western HRP substrate (Merck Millipore, Burlington, MA, USA) and the images were acquired using Alliance 9.7 Western Blot Imaging System (UVItec Limited, Cambridge, UK). In-house made 1:1000 mouse polyclonal antibody anti-tubulin and 1:1000 mouse monoclonal antibody anti-p84 (Abcam, Cambridge, UK) were used as cytoplasmic and nuclear controls, respectively (Ayala et al., 2008).

### RNAseq and analysis of differentially expressed genes (DEGs)

Total RNA was extracted from luciferase (control), hnRNP Q and hnRNP R depleted SH-SY-5Y cells, as described previously. RNA sequencing was performed by Eurofins (www.eurofins.com) using Illumina HiSeq 2500 instrument. Data processing was carried out with the following software: HiSeq Control Software v2.0.12.0, RTA v1.17.21.3 and bcl2fastq-1.8.4. Alignment to human reference sequence was performed by BWA-MEM (version 0.7.12-r1039, http://bio-bwa.sourceforge.net/) and the raw read counts were created using featureCounts (http://bioinf.wehi.edu.au/featureCounts/). Only reads with unique mapping positions and a mapping quality score at least 10 were considered for read counting. Raw read counts were converted to Counts per million (CPM) values by Trimmed mean of M-values (TMM) normalization (edgeR package http://bioconductor.org/packages/release/bioc/html/edgeR.html, (Robinson and Oshlack, 2010). Features had to have a counts-per-million value of more than one in at least three samples or were removed, resulting in the removal of 47,622 of the 64,769 features. Differential expression analysis was performed on the remaining 17,417 genes using edgeR package. GOseq package from R (Young et al., 2010) was also used for Gene ontology (GO) and KEGG pathway analysis. Categories significantly enriched (*p*-value < 0.05) were considered.

### Accession numbers

The data discussed in this publication have been deposited in NCBI's Gene Expression Omnibus (Edgar et al., 2002) and are accessible through GEO Series accession number GSE114165. [The following secure token has been created to allow review of record GSE114165 while it remains in private status: kjubwgcqjjyljct].

## Results

### Different subcellular localization of human hnRNP Q and hnRNP R in the neuroblastoma SH-SY5Y cell line

In our previous study, we have used *Drosophila melanogaster* as a model organism to study the effects of hnRNP depletion in a model of TDP-43/TBPH gain- and loss-of-function and we identified CG17838/Syncrip (Syp) (Figure 1A) as a potentially very powerful modulator of TDP-43 effects (Appocher et al., 2017). Interestingly, of the two human Syp orthologs (hnRNP Q and hnRNP R), only hnRNP Q was shown to be able to rescue missplicing effects due to TDP-43 silencing.

To better characterize the roles played by human hnRNP Q and hnRNP R in the neuronal-like cell line SH-SY5Y, we first investigated their subcellular localization by carrying out immunofluorescence (IF) staining for the endogenous proteins. In these cells, hnRNP Q showed punctate localization both in nucleus and cytoplasm whilst the hnRNP R IF signal was predominantly nuclear (Figure 1B). In particular, the presence of the cytoplasmic variant of hnRNP Q in granule-like structures further supports previous results showing the involvement of hnRNP Q in mRNA trafficking (Bannai et al., 2004; Chen et al., 2012).

Western blot analysis of nuclear and cytoplasmic fractions was also carried out to confirm these results and check for isoform production.

Regarding hnRNP Q, three major splicing isoforms of this protein have been so far previously reported (Mourelatos et al., 2001). These isoforms are characterized by the presence of two NLS in the hnRNP Q3 and hnRNP Q2 variants (with a molecular weight of 65 kDa and 70 kDa, respectively) and one NLS in the hnRNP Q1 (with a molecular weight of 62 kDa) (Figure 1A). Three immunoreactive bands (~ 58, ~66, and ~ 75 kDa) were detected by Western blot analysis, differentially distributed between nucleus and cytosol (Figure 1C). The molecular weight of ~ 75 kDa is consistent with that of hnRNP Q2/hnRNP Q3 isoforms and the molecular weight of ~ 66k Da with that of hnRNP Q1 isoform. The apparent molecular weight of the lower band (~58 kDa) cannot be associated to any known hnRNP Q isoform and could be corresponding to a further variant that still remains to be characterized.

The same analysis was repeated for hnRNP R, confirming its presence predominantly in the nuclear fraction (Figure 1C). The antibody used for staining the membrane (ab30930) detected three bands: ~71, ~75, and ~80 kDa. According to literature the major hnRNP R isoform, also known as R1 (NP_005817.1. NM_005826.4. [O43390-1]) presents a molecular weight of ~80 kDa whilst a second characterized variant, namely R2 (NP_001284549.1, NM_001297620.1 [O43390-3]) presents a molecular weight of ~75 kDa (Hassfeld et al., 1998; Huang et al., 2005). According to these data, we concluded that our immunoreactive bands of ~75 kDa and ~80 kDa were R2 and R1, respectively. On the other hand, the band of ~71 kDa could be another splicing variant of hnRNP R that still needs to be identified.

**Figure 2:**
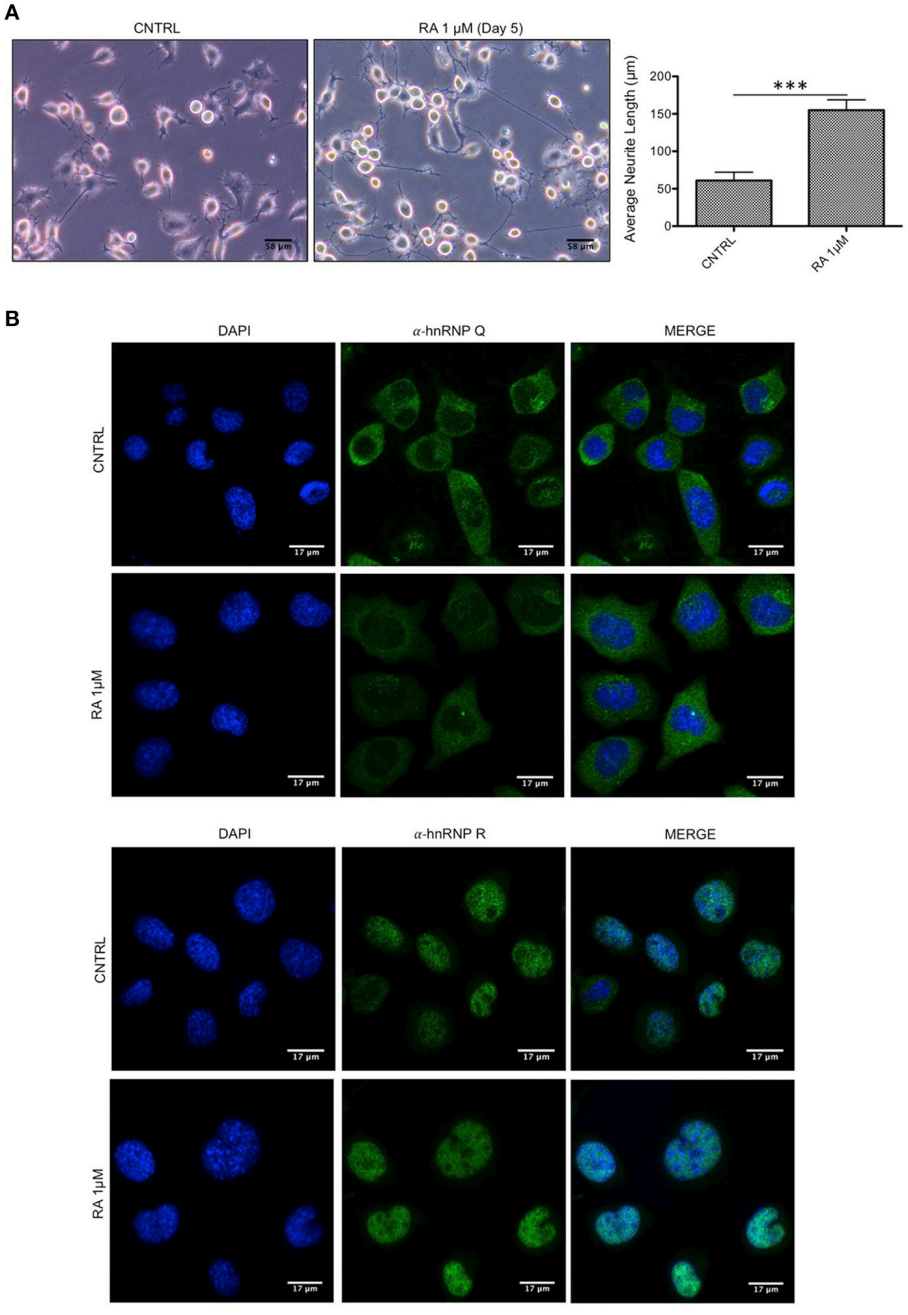
Endogenous localization of hnRNP Q and hnRNP R is not affected by differentiation in NSC-34 cells. **(A)** Light microscopy images of undifferentiated (CNTRL) and differentiated cells treated up to 5 days with medium containing 1 μM of all-trans retinoic acid (RA 1 μM). Scale bars: 58 μm, 20X magnification. Neurite length comparison (gray bars) of CNTRL and RA is also reported as mean ± standard error. Statistical differences were evaluated using t-students (***p < 0.001). **(B)** Immunofluorescence analysis of the endogenous mouse hnRNP Q and hnRNP R (shown in green) in NSC-34 cells with or without 1 μM RA treatment. Nuclei were visualized using DAPI staining. Scale bars: 17 μm.

Subsequently, we tested if hnRNP Q or hnRNP R might change their cellular localization after neuronal differentiation. To this aim, considering the extremely high conservation of these two proteins in mouse (more than 99% identity and similarity), immunofluorescence experiments were carried out after inducing differentiation of the murine motoneuron-like NSC-34 cell line, due to their ability to differentiate in more neuron-like cells (Figure 2A). The staining showed that hnRNP Q and hnRNP R did not change their subcellular distribution after differentiation (Figure 2B).

Overall, the different localization of hnRNP Q and hnRNP R suggests that their nuclear-cytoplasmic distribution is differentially regulated and this could be reflected in a differential control of cellular pathways.

### Knockdown of hnRNP Q and hnRNP R affects the expression of genes related to brain functions, neurodegeneration and inflammatory response

Following this immunolocalization analysis, we therefore decided to analyze the whole transcriptome status of SH-SY5Y silenced for these hnRNPs in order to identify the genes whose expression is commonly or differentially regulated by these proteins.

**Figure 3:**
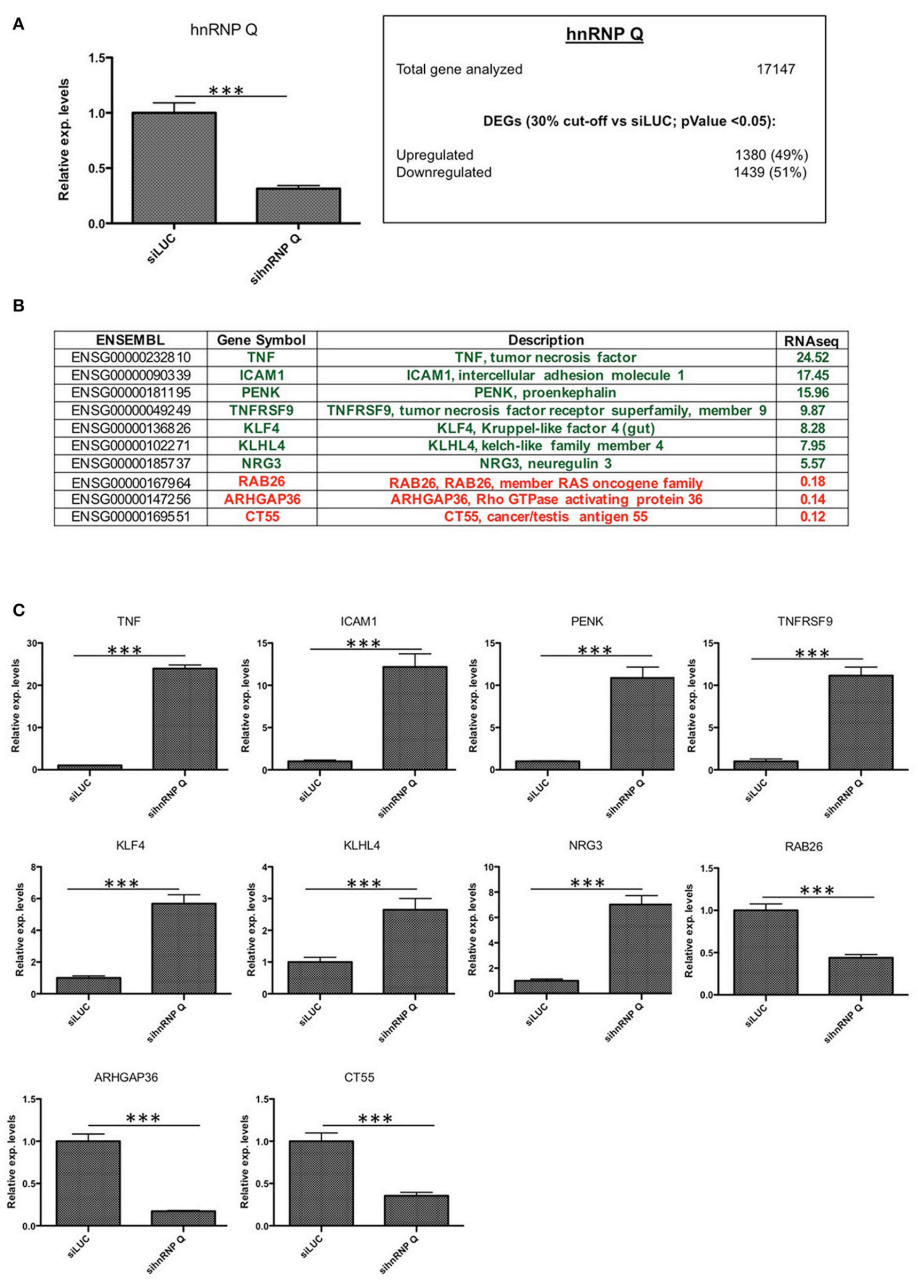
Validation of hnRNP Q silencing and comparison between RNA-seq and RT-qPCR results. **(A)** Assessment of hnRNP Q siRNA silencing efficiency using qPCR (***p < 0.001) and summary of differentially expressed genes (DEGs with *p* < 0.05) with number of downregulated (<0.7x vs. siLUC) and upregulated (>1.3x vs. siLUC) genes after sihnRNP Q treatment. **(B)** List of DEGs validated by RT-qPCR and associated with brain functions/neurodegeneration (PENK KLF4, KLHL4, NRG3, RAB26, and ARHGAP36), inflammation (TNF, ICAM1, TNFRSF9), and other functions (CT55). **(C)** RT-qPCR validation of 10 selected transcripts. Each bar reports mean ± standard error of three independent experiments. Statistical differences were evaluated using t-students (*p < 0.05, **p < 0.01, ***p < 0.001).

**Figure 4:**
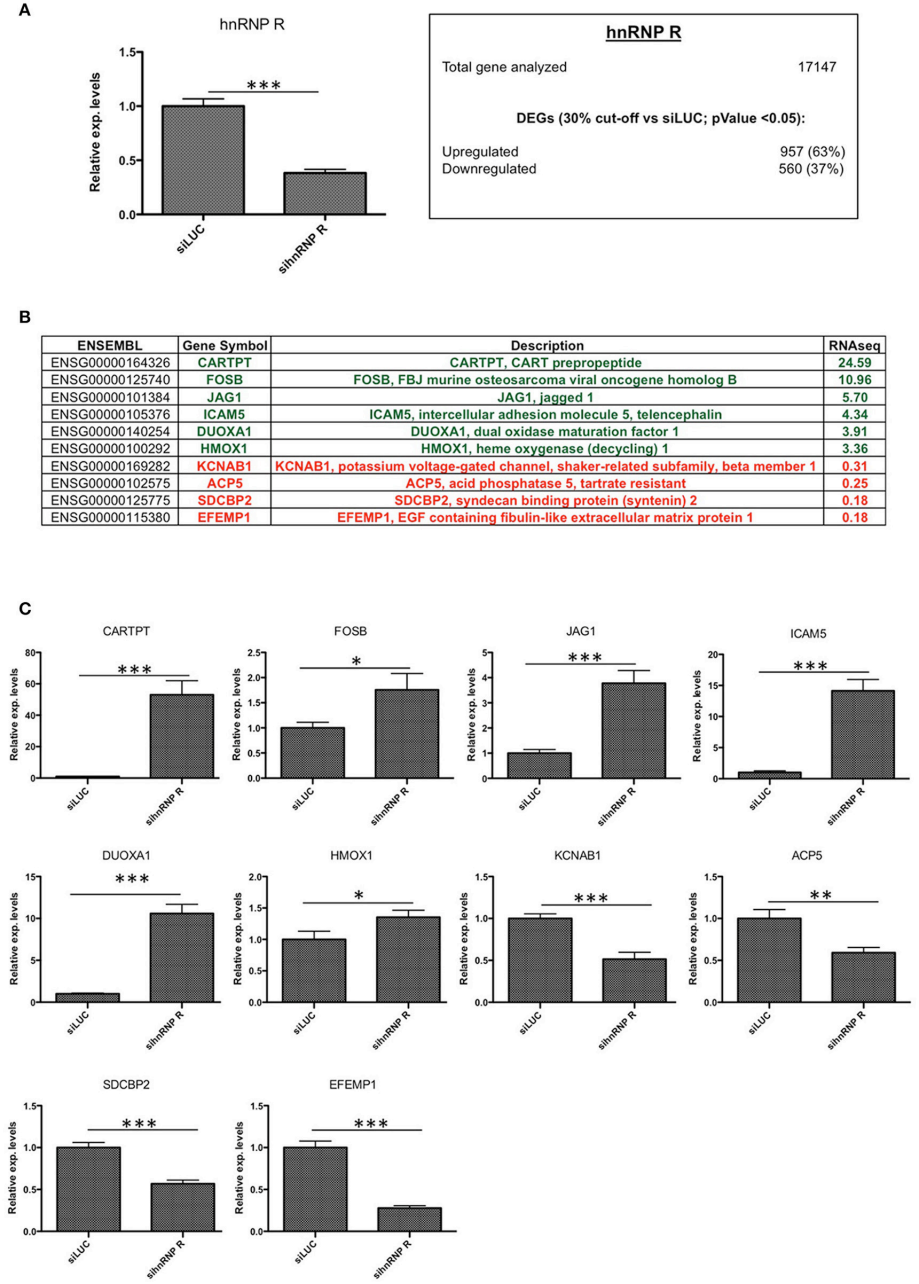
Validation of hnRNP R silencing and comparison between RNA-seq and RT-qPCR results. **(A)** Assessment of hnRNP R siRNA silencing efficiency using qPCR (***p < 0.001) and summary of differentially expressed genes (DEGs with p < 0.05) with number of downregulated (<0.7x vs. siLUC) and upregulated (>1.3x vs. siLUC) genes after sihnRNP R treatment. **(B)** List of DEGs validated by RT-qPCR and associated with brain functions/neurodegeneration (CARTPT, FOSB, JAG1, DUOXA1, HMOX1, KCNAB1, SDCBP2, EFEMP1), inflammation (ICAM5, ACP5). **(C)** RT-qPCR validation of 10 selected transcripts. Each bar reports mean ± standard error of three independent experiments. Statistical differences were evaluated using t-students (*p < 0.05, **p < 0.01, ***p < 0.001).

**Figure 5:**
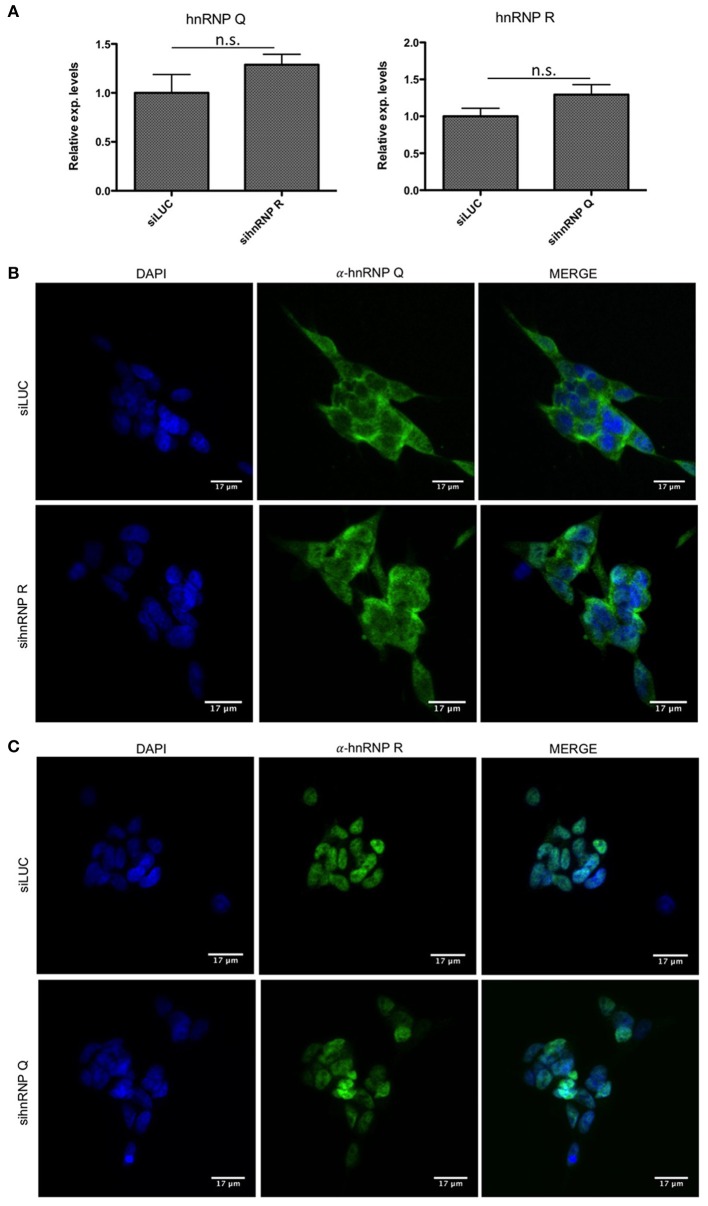
mRNA levels and cellular localization of endogenous hnRNP Q and hnRNP R after knockdowns in SH-SY5Y cells. **(A)** Relative expression of hnRNP Q after siRNA against hnRNP R (sihnRNP R) and vice-versa. All the samples were compared to siLUC treated cells. Each bar reports mean ± standard error of three independent experiments. Statistical differences were evaluated using t-students (ns: not significant). **(B)** Immunofluorescence analysis of the endogenous human hnRNP Q (shown in green) in SH-SY5Y cells after siRNA treatment against hnRNP R. Nuclei were visualized using DAPI staining. Scale bars: 17 μm. **(C)** Immunofluorescence analysis of the endogenous human hnRNP R (shown in green) in SH-SY5Y cells after siRNA treatment against hnRNP Q. Nuclei were visualized using DAPI staining. Scale bars: 17 μm.

First of all, we checked the downregulation of hnRNP Q and hnRNP R using qPCR (Figure 3A, 4A) and then we investigated if the silencing of hnRNP Q was able to affect the gene expression levels and cellular localization of hnRNP R and vice-versa (Figure 5). We observed a significant reduction of mRNA levels of hnRNP Q and hnRNP R after their silencing. Moreover, we observed no significant differences in the mRNA levels as well as in the endogenous localization of hnRNP Q after sihnRNP R and of hnRNP R after sihnRNP Q.

Then, we carried out an RNA-seq analysis of three independent knockdowns for each hnRNP and the putative differentially expressed genes (DEGs) were identified comparing the data of hnRNP Q or hnRNP R silencing to siLUC control samples. To identify up- and downregulated genes, the cut-off values used were the fold change (FC) value (upregulation cut-off: >1.3; downregulation cut-off: <0.7-FC) and the *p*-value < 0.05. Following silencing of hnRNP Q, a total of 2,819 genes (out of the 17,147 analyzed genes) resulted to be differentially expressed. These included 1,380 (49%) upregulated and 1,439 (51%) downregulated genes (Figure 3A). On the other hand, following silencing of hnRNP R, 1517 genes (out of the 17,147 analyzed genes) were differentially expressed, 957 (63%) upregulated and 560 (37%) downregulated (Figure 4A).

In order to validate these RNA-seq data, we monitored by qPCR the expression (after hnRNP Q or hnRNP R silencing) of 10 genes selected among the top 100 differentially expressed genes (Figures 3B, 4B). We selected these genes considering their potential involvement in neuron development/functions as well as neuroinflammation.

Regarding cells silenced for hnRNP Q, tumor necrosis factor (TNF), intercellular adhesion molecule 1 (ICAM1), proenkephalin (PENK), tumor necrosis factor receptor superfamily, member 9 (TNFRSF9), Kruppel-like factor 4 (gut) (KLF4), kelch-like family member 4 (KLHL4), and neurogulin 3 (NRG3) were found to be upregulated, while RAB26, member RAS oncogene family (RAB26), Rho GTPase activating protein 36 (ARHGAP36) and cancer/testis antigen 55 (CT55) were found downregulated (Figure 3C). On the other hand, concerning cells silenced for hnRNP R, CART prepropetide (CARTPT), FBJ murine osteosarcoma viral oncogene homolog B (FOSB), jagged 1 (JAG1), intercellular adhesion molecule 5, telencephalin (ICAM5), dual oxidase maturation factor 1 (DUOXA1) and heme oxygenase (decycling 1) (HMOX1) were found to be upregulated, while potassium voltage-gated channel, shaker-related subfamily beta (KCNAB1), acid phosphatase 5, tartrate resistant (ACP5), syndecan binding protein (syntenin) 2 (SDCBP2), and EGF containing fibulin-like extracellular matrix protein 1 (EFEMP1) were found to be downregulated (Figure 4C). In conclusion, the results of our qPCR validation are consistent with those obtained with the RNA-seq analysis.

**Figure 6:**
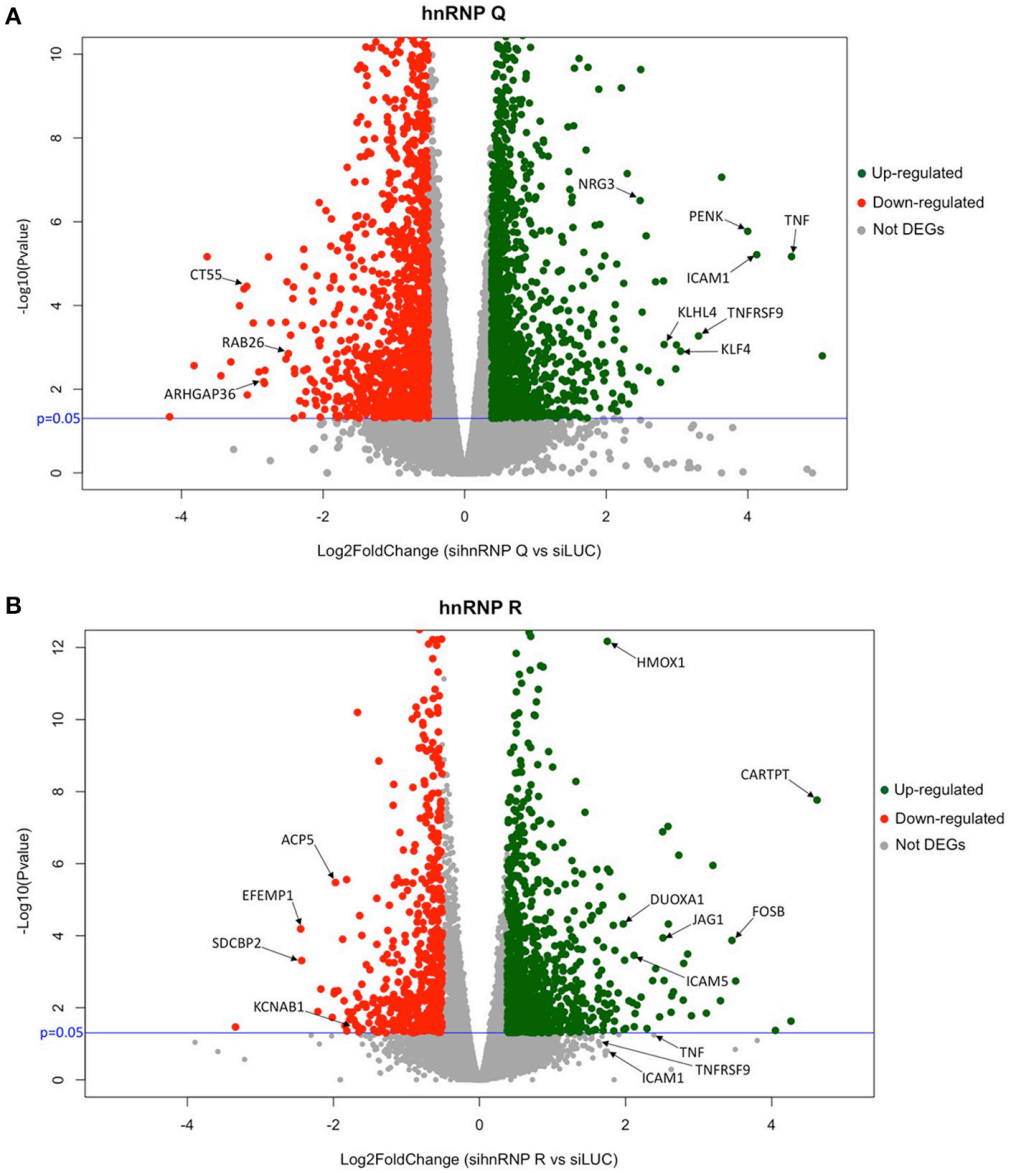
Volcano plot of hnRNP Q and hnRNP R. Schematic representation of RNA-seq data of sihnRNP Q **(A)** and sihnRNP R **(B)** treated cells. Up- and downregulated genes are reported as green and red dots, respectively. Not DEGs are represented as gray dots. Black arrows show RT-qPCR validated genes. *P* threshold (=0.05) is reported in blue.

Volcano plots were also used to obtain a general overview the results obtained in both sihnRNP Q and sihnRNP R treated cells (Figure 6). Differentially expressed genes are highlighted in red (downregulated) and in green (upregulated) based on the *p*-value and FC variation with respect to the control treated cells (siLUC). In this diagram, we also report the position of the DEGs validated in Figures 2C, 3C using RT-qPCR.

### Gene ontology (GO) enrichment and KEGG pathway analysis reveal different and common features regulated by hnRNP Q and hnRNP R

**Figure 7:**
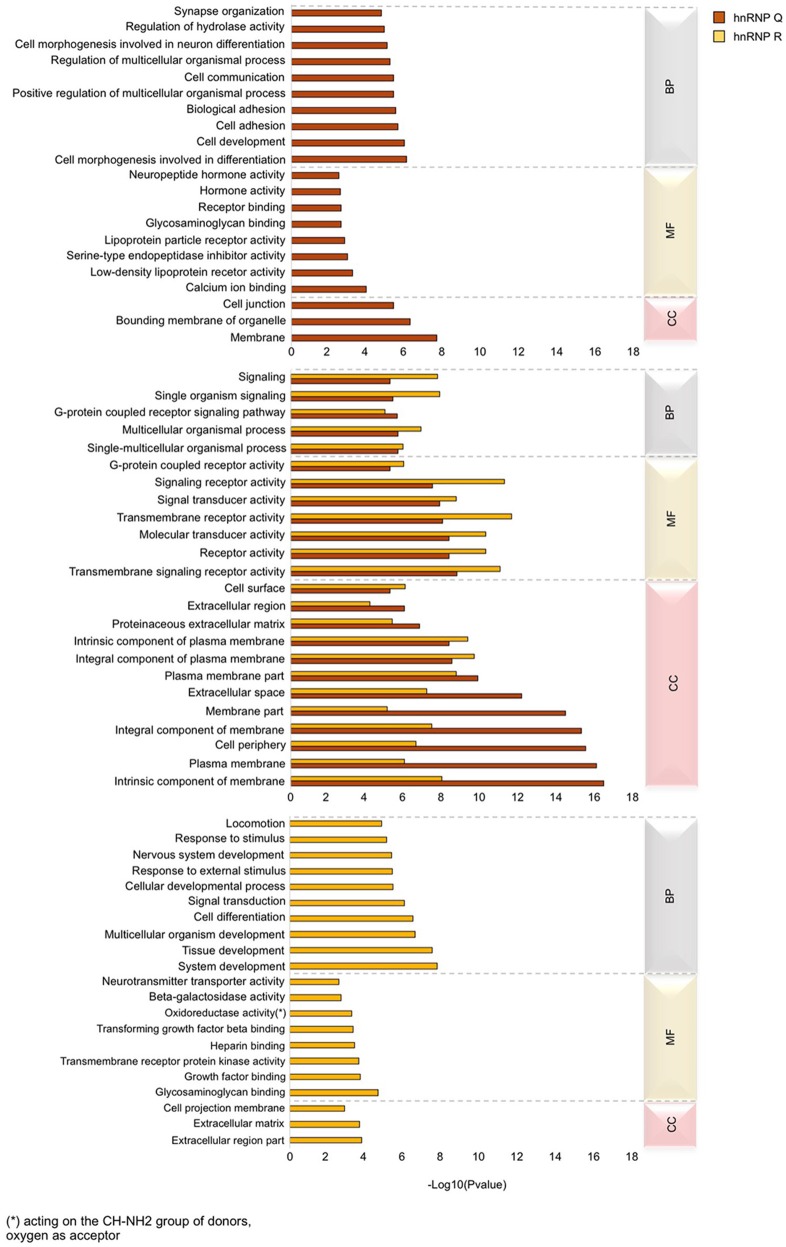
Gene ontology (GO) analysis of DEGs in sihnRNP Q and sihnRNP R treated cells. GO enrichment analysis was performed using GOseq package Three major GO categories are highlighted: biological process (BP), molecular function (MF), and cellular component (CC). The first fifteen sub-categories (*p* < 0.05) of DEGs of each category are reported for both sihnRNP Q and sihnRNP R treated cells.

We next carried out enrichment analysis to find which GO terms are over-represented in the genes regulated by hnRNP Q and hnRNP R, in order to highlight differences and similarities in specialization between these two orthologs. To this aim, we took advantage of the GOseq R Bioconductor package (Young et al., 2010) and considered for the final analysis only GO term of the “biological process” (BP), “molecular function” (MF), and “cellular component” (CC) categories reaching the *p*-value threshold < 0.05 for significance. For both hnRNP Q and hnRNP R, the top 25 GO terms of the major three categories were selected and sorted by their presence or absence in each hnRNPs. This approach led us to define categories specific for hnRNP Q or hnRNP R and categories commonly present in both two proteins (Figure 7).

**Figure 8:**
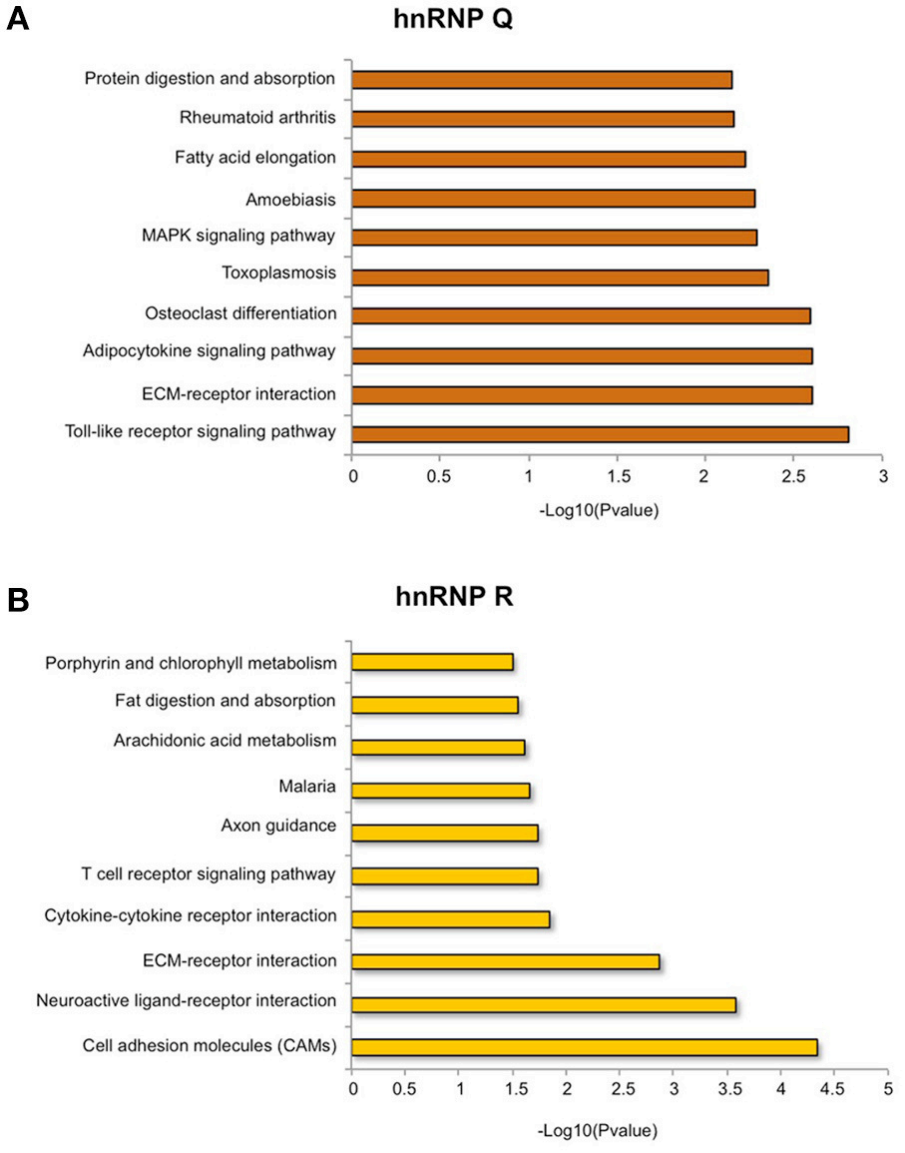
KEGG pathway analysis of DEGs in sihnRNP Q and sihnRNP R treated cells. First ten KEGG pathway identified (*p* < 0.05) in DEGs in SH-SY5Y cells depleted for hnRNP Q **(A)** and hnRNP R **(B)**. KEGG pathway analysis was performed using GOseq package.

Regarding hnRNP Q, out of 2819 DEGs used as input for GO analysis, we identified a total of 1,152 terms with significant gene enrichment. The top enriched GO categories were “membrane” (*p*-value = 3.17E-08), “bounding membrane to organelle” (*p*-value = 7.29E-07), “cell morphogenesis involved in differentiation” (*p*-value = 1.10E-06), “cell development” (*p*-value = 1.42E-06) and “cell adhesion” (*p*-value = 3.17E-06). On the other hand, for hnRNP R, out of 1,517 DEGs used as input for GO analysis, we identified a total of 955 terms with significant gene enrichment. The top enriched GO categories were “system development” (*p*-value = 2.34E-08), “tissue development” (*p*-value = 4.21E-08), “multicellular organism development” (*p*-value = 3.39E-07), “cell differentiation” (*p*-value = 4.28E-07) and “signal transduction” (1.13E-06). Notably, when we looked at GO terms differentially enriched in hnRNP Q and hnRNP R DEGs, we found that “intrinsic component of membrane,” “plasma membrane,” “cell periphery,” “integral component of membrane,” and “membrane part” were particularly enriched in hnRNP Q depleted cells, while “signal receptor activity,” “transmembrane receptor activity,” “transmembrane signaling receptor activity,” “receptor activity,” and “molecular transducer activity” were more enriched in hnRNP R depleted treated cells. Furthermore, we also looked at the KEGG pathway analysis using GOseq package from R. We found 29 and 16 terms with significant gene enrichment (*p*-value < 0.05) for hnRNP Q and hnRNP R, respectively (Figure 8). In particular, we noticed that most of the pathways identified by KEGG pathways analysis for both these two proteins were related to inflammation. Indeed, “toll-like receptor signaling pathway” (*p*-value = 0.002), “ECM-receptor interaction” (*p*-value = 0.002), “adipocytokine signaling pathway” (*p*-value = 0.003), “toxoplasmosis” (*p*-value = 0.004) and “rheumatoid arthritis” (*p*-value = 0.007) were particularly enriched in DEGs obtained by sihnRNP Q silencing, whereas “cell adhesion molecules (CAMs)” (*p*-value = 4.58E-05), “ECM-receptor interaction” (*p*-value = 0.001), “cytokine-cytokine receptor interaction” (*p*-value = 0.01), “T cell receptor signaling pathway” (*p*-value = 0.02) and “malaria” (*p*-value = 0.02) were particularly enriched in DEGs obtained by sihnRNP R silencing.

In conclusion, this analysis shows that hnRNP Q and hnRNP R have presumably acquired different functional specialization during evolution. Our results suggest that hnRNP Q plays a role in the assembly of plasma membrane lipid layers and organelles as well as in the regulation of events associated with cell-cell and cell-extracellular matrix contacts. On the contrary, hnRNP R seems to be implicated mostly in processes associated with differentiation and development of cells/tissues, as well as cell signaling.

## Discussion

The elucidation of the molecular mechanisms underlying RNA regulation in both physiological and pathological processes is hampered by the great complexity of RBP networks, that can occur through the establishment of highly specific or loosely-specific interactions (Liachko et al., 2010; Cohen et al., 2015) and their post-translational modifications (Dassi, 2017).

To fill this gap, we focused our attention on two prominent but less studied members of the hnRNP family, hnRNP Q, and hnRNP R.

Previous studies have shown that hnRNP Q has multiple functions in mRNA metabolism, ranging from pre-mRNA splicing to mRNA editing, stability control, transport, and translation (Blanc et al., 2001; Bannai et al., 2004; Chen et al., 2008; Weidensdorfer et al., 2009; Kim et al., 2010). On the other hand, hnRNP R, a highly hnRNP Q related hnRNP, seems to be implicated in processing and localization of β-actin mRNA by binding its 3′ UTR in motor axons (Rossoll et al., 2003). In addition, it has been suggested that hnRNP Q and hnRNP R cooperates in regulating cytoplasmic mRNA trafficking (Mourelatos et al., 2001; Rossoll et al., 2002).

More recently, it has been confirmed that hnRNP R interacts with the 3′ UTR of mRNAs (Briese et al., 2018) and that, along with its main interactor, the noncoding RNA 7SK, it seems to coregulate the axonal transcriptome of motoneurons (Briese et al., 2018).

Therefore, despite recent progresses in the understanding of hnRNP Q and hnRNP R functions, little is about their roles in regulation of gene expression and about the potential targets of their actions. Looking at the transcriptome status of SH-SY5Y cells silenced with siRNA against hnRNP Q and hnRNP R, we found distinctive and common features associated to DEGs in both these proteins. Moreover, in the top 100 DEGs of both proteins we identified an important subset of genes that correlate with neurodegeneration and inflammation cellular pathways.

In general, regarding hnRNP Q, our study suggests that this factor can regulate predominantly the expression of genes potentially impacting the immune response and inflammation (Figure 8A). In fact, the two immune-related KEGG pathways “Rheumatoid arthritis” and “Toxoplasmosis” were found to be enriched in hnRNP Q DEGs. Indeed, it was observed that infection of Toxoplasma gondii is associated with neuronal impairment and inflammation in mice and humans (Carruthers and Suzuki, 2007) and the inhibition of TNF signaling in patients suffering from rheumatoid arthritis is protective against Alzheimer's disease (Steeland et al., 2018). Finally, it is worth noting that several lines of evidence support a role for Toll-like receptors (TLRs) in the pathogenesis of neurodegenerative diseases, such as ALS (Casula et al., 2011), Alzheimer's disease (Reed-Geaghan et al., 2009) and in multiple sclerosis (Prinz et al., 2006; Marta et al., 2008).

On the other hand, regarding hnRNP R, the KEGG pathway analysis suggests that this protein predominantly influences the expression of genes related with brain functions and inflammation (Figure 8B). In fact, two neuronal-related KEGG pathways (“Axon guidance” and “Neuroactive-ligand receptor interaction”) and immune/inflammation-related KEGG pathways (“T receptor signaling”, “Cytokine-cytokine receptor interaction”, “Cell adhesion molecules” “ECM-receptor interaction,” and “Arachidonic acid metabolism”) were found to be enriched in hnRNP R DEGs.

Then, taking a closer look at the regulated genes, regarding hnRNP Q, we found KLF4 (Qin and Zhang, 2012), NRG3 (Zhou et al., 2018), and PENK (Ernst et al., 2010) up-regulated following hnRNP Q silencing. In particular, NRG3 and PENK encode two neuronal proteins associated with synapse plasticity and neuronal disorders, respectively. The silencing of hnRNP Q was also able to down-regulate both RAB26 and RAB33B, that have been shown to bind to ATG16L1 in the fly neuromuscular junctions, thus suggesting the implication of Syncrip in recycling of synaptic vesicle proteins through the autophagy pathway (Binotti et al., 2015). This observation is particularly intriguing because of the role played by TDP-43 and its fly ortholog TBPH in the neuromuscular junction formation. In fact, a previously generated TBPH-null allele Drosophila ALS model showed specific alterations of neuromuscular junctions (Feiguin et al., 2009; Godena et al., 2011; Langellotti et al., 2016; Romano et al., 2018) and the hTDP43A315T transgenic mouse model of ALS presented a strong reduction of synaptic vesicles in the NMJs (Magrané et al., 2013).

Finally, neuronal expression has been reported for both KLHL4 and ARHGAP36, although their neuronal function or possible connection with diseases is still not fully elucidated (Braybrook et al., 2001; Rack et al., 2014).

Regarding hnRNP R, we observed up-regulation of CARTPT, FOSB, JAG1, DUOXA1 and HMOX1 and down-regulation of KCNAB1, SDCBP2, and EFEMP1. It is interesting to note that CARTPT encodes a prepropeptide acting as a neurotransmitter in association with GABA (g-aminobutyric acid) (Smith et al., 1999) and substance P (Hubert and Kuhar, 2005). Furthermore, the maturation factor of NADPH oxidase Dual oxidase 1 (DUOXA1) was found to promote neuronal-like differentiation of p19 embryonal carcinoma cells following p53 expression (Ostrakhovitch and Semenikhin, 2011). Interestingly, JAG1 and, in a more general view, the Notch signaling pathway are important for the spatial memory and their expression is altered in the hippocampus of people suffering from Alzheimer's disease (Marathe et al., 2017). In addition, HMOX1 expression is also up-regulated in neurons and astrocytes derived from hippocampus, cerebral cortex, and subcortical white matter of Alzheimer's patients (Schipper et al., 1995).

On the other hand, due to the increase importance of the inflammatory response in the pathogenesis of neurodegenerative disease (Wyss-Coray and Mucke, 2002; Block and Hong, 2005; Glass et al., 2010), it was particularly interesting to note that cells depleted by hnRNP Q and hnRNP R showed a prominent disruption of this pathway. In particular, the inflammatory proteins TNF, TNFRSF9, and ICAM1 were upregulated by the silencing of hnRNP Q, likewise to what observed with silencing of TDP-43 and DAZAP1 (Appocher et al., 2017). TNF is a pro-inflammatory cytokine that is expressed in the central nervous system and its soluble form can promote neuronal inflammation, occurring in neurodegenerative conditions such as ALS, multiple sclerosis, Alzheimer's and Parkinson's diseases (McCoy and Tansey, 2008). TNFRSF9, also known as CD137, is a member of the tumor necrosis factor receptor family that was demonstrated to promote the oligodendrocyte apoptosis when bound to its ligand, through the release of reactive oxygen species. Finally, ICAM1 was found to be overexpress in age-dependent neurodegeneration and localized in amyloid plaques of Alzheimer's patients (Miguel-Hidalgo et al., 2007).

By contrast, in sihnRNP R treated cells, other two immune-related proteins (namely, ICAM5 and ACP5) were found to be differentially regulated, while ICAM1, TNF, and TNFRSF9 were not significantly altered. In particular, ICAM5 (telencephalin) has been described to mediate the neuroprotective effects by inhibiting the pro-inflammatory cascade of ICAM1 (Tian et al., 2008). Regarding ACP5, the presence of this gene was detected in brain and spinal cord of rat and the connection with inflammation lies in the abnormal macrophage response to bacteria in mice lacking of this enzyme (Bune et al., 2001).

In conclusion, regarding the possible molecular mechanisms by which depletion of hnRNP Q and hnRNP R influence the gene expression profiles of SH-SY5Y, we are tempted to speculated that that hnRNP R and hnRNP Q might regulate the abundance of transcripts by affecting the mRNA stability through their interaction with the 3′UTR. This hypothesis is supported by the known ability to bind the 3′ UTRs of mRNAs. However, other mechanisms (such as alternative splicing associated to NMD or alternative splicing regulation of transcription factors) can be implicated and cannot be excluded at the present stage. Nonetheless, our study sheds light on the distinctive functions of hnRNP Q and hnRNP R in human neuronal cells and, in general, provides insights on the involvement of the hnRNP family in controlling neuronal and inflammatory pathways, strengthening the hypothesis that differential expression of these RBPs could play an essential role in modulating the onset and progression of neurodegenerative disorders.

## Author contributions

SC and MR performed the experiments. EB and MR designed the study. SC, MR, and EB analyzed the results and wrote the manuscript.

### Conflict of interest statement

The authors declare that the research was conducted in the absence of any commercial or financial relationships that could be construed as a potential conflict of interest.
